# Annotations

**Published:** 1887-03-26

**Authors:** 


### ANNOTATIONS.

**Faith Healing.** Some weeks ago we published a leading article on the subject of Faith Healing, to which public attention had just been once more directed by the utterances of Mr. Milner Stephen. In that article, as our readers may recollect, we somewhat briefly, but as we think exhaustively, discussed the subjeet from the point of view both of the uninstructed and also of the educated and thoughtful man, with the result that we could not, nor can we now, discover that there is either possibility or proof of any of the so-called miracles of the present day having taken place. We said: "If a wonderful work has been done, it must be such that no man can deny or doubt it. *If a blind man has been cured, it must be evident to all the world that he now can see.*" We have purposely italicised this last sentence, and for what reason our readers will presently learn. In a case of faith-healing recently reported in *The Daily Chronicle*, the Lincoln correspondent of that journal states that one Richard Green, a native of Lincoln now residing in Nottingham, has, "owing to an attack of paralysis, followed by epileptic fits, for the past six years been unable to get about except with the aid of crutches, and *could not see without spectacles*." The correspondent of *The Daily Chronicle* proceeds to state that Green has during the past month attended faith-healing meetings in Nottingham, and has been wonderfully and mysteriously cured. Moreover, Green, now paying a visit to his parents in Lincoln, has addressed a meeting of the Salvation Army, and, to the amazement of those who knew his former lamentable condition, he was full of life and spirits, and walked as if nothing had ever been amiss with him. There is no specific statement of his having discarded spectacles, but from their former mention we may presume that he has done so. This account produced a letter from the somewhat notorious Dr. Forbes Winslow, who apparently gives full credit to the story, and states that a similar recovery has been manifested at Nice, during the late earthquakes, in the case of "a relative of one of our late Cabinet Ministers," to whom the sudden fright restored the use of limbs apparently stricken by paralysis. Passing over the noteworthy fact, that in neither of these two cases does the writer vouch for the truth of his statement, or say "*I* know the case to be so," and "*I* have seen the cure," we would ask the correspondent of *The Daily Chronicle*, "What has become of Richard Green's spectacles?" Will any two, will even one credible witness, of position and honour beyond dispute, come forward and say that he knew Richard Green to be dependent upon spectacles for, let us say, the reading of his Bible, and that now he can read fluently without them at whatever chapter the book may be opened? Will Dr. Forbes Winslow give the name of the "relative of one of our late Cabinet Ministers," and can he certify that the case was really one of paralysis? We are far more willing to believe in the effect of a sudden shock of earthquake upon a nervous and hypochondriacal person, than in the cure of paralysis, epilepsy, and dimness of vision of six years' standing under the circumstances described. If these are genuine nineteenth century miracles, by all means let proof of the fact be forthcoming, otherwise judgment must go by default.

**Alleged Inhumanity at Guy's.** Most readers will have seen the accounts in the daily papers of the man whose fractured fibula was put up in plaster-of-paris at Guy's, whilst the man himself was not allowed to become an in-patient. A letter recording a similar case in private practice appears elsewhere. The matter is only referred to here for the purpose of expressing sympathy with Dr. Steele, under a somewhat unpleasant public misapprehension. It is well for the hospitals that they should feel themselves exposed to the Argus eyes of the newspapers. Happily, in many respects they are well able to bear the light of day. So far as skilful treatment and kindness are concerned, there is practically nothing to be desired in the London hospitals as a whole. Such are the conditions of life and duty at those institutions, as Dr. Potter states in another column, that it is practically impossible for either unkindness or unskilfulness ever to take place. With regard to Dr. Steele himself, anyone who knows him will laugh at the idea of his turning any patient out who was in the least need of a bed within the hospital walls. If he errs at all, it will certainly not be on the side of want of consideration for the poor.

